# Acute Postoperative Intestinal Obstruction Due to Abdominal Drains: A Case Report

**DOI:** 10.7759/cureus.105257

**Published:** 2026-03-15

**Authors:** Mohammed Doumar, Abdelhakim Harouachi, Badr Serji

**Affiliations:** 1 Surgical Oncology, Centre Hospitalier Universitaire Mohammed VI Oujda, Oujda, MAR; 2 Surgical Oncology, Faculty of Medicine and Pharmacy, Mohamed I University, Oujda, MAR

**Keywords:** abdominal surgery, drain, intestinal obstruction, jackson-pratt drain, postoperative complications

## Abstract

Early small bowel obstruction caused by a drain in the postoperative period is a rare complication, with few cases reported in the literature. Its diagnosis is difficult, and its complications can be severe. The objective of this case report is to describe the clinical and radiological features suggestive of intestinal occlusion associated with abdominal drains and to summarize the management strategies reported in the literature for this rare complication. We report a case of acute intestinal obstruction caused by an abdominal drain after rectal surgery, documented in the oncology surgery department of the Regional Oncology Hospital of Oujda, Morocco, in 2023. Our report describes the case of a 64-year-old woman who developed acute intestinal obstruction following rectal surgery, caused by a prophylactic Jackson-Pratt-type drain. Paraclinical investigations revealed a mechanical small bowel obstruction with a sudden change in bowel caliber. Data from the literature show that intestinal obstructions caused by drains are rare but serious. Interpreting CT signs in a postoperative context is complex. Rigorous management of drain placement indications could reduce the incidence of obstructions related to these devices. Early small bowel obstruction as a complication of an abdominal drain is rare. Its clinical presentation is misleading and may be masked by seemingly normal signs after surgery, such as postoperative ileus. However, it should be considered as a differential diagnosis in cases of postoperative bowel obstruction. Once the diagnosis is made and the absence of signs of digestive compromise requiring reoperation has been confirmed, the drain should be removed.

## Introduction

Intestinal obstruction is a common complication following abdomino-pelvic surgery. This type of surgery is now well recognized as a risk factor for intestinal obstruction in the short, medium, and long term (the incidence of reoperation for adhesive bowel obstruction after abdomino-pelvic surgery is 2.5%) [1]. These intestinal obstructions encompass a range of clinical scenarios, but they are most often grouped under a clinical occlusive syndrome, which includes several elements: cessation of stool and gas, nausea or vomiting, abdominal pain, and abdominal distension [2,3].

Clinical suspicion prompts the indication for urgent computed tomography (CT) imaging, which usually includes, unless contraindicated, two acquisitions (non-contrast and portal venous phase contrast-enhanced). Among these obstructions, the early post-operative period represents a particular situation. Some elements that usually guide decision-making are difficult to interpret. Clinical examination and laboratory tests are frequently altered in the postoperative setting. Abdominal pain and a reactive inflammatory syndrome following surgery are common and may not have pathological significance. Similarly, the interpretation of abdomino-pelvic CT in the early postoperative period is challenging. Pneumoperitoneum or infiltration of abdominal fat caused by the surgical approach must be interpreted with caution in this context, without necessarily indicating pathology [3].

Drainage is an essential step in colorectal surgery when indicated, but it can lead to potentially serious complications. Understanding these complications is crucial for prevention, early diagnosis, and appropriate management. This rare case, seldom reported in the literature, highlights the need to consider this diagnosis and distinguish it from the main differential diagnosis, which is postoperative paralytic ileus.

The objectives of our manuscript are to describe the clinical and radiological features suggestive of bowel obstruction associated with abdominal drains and present the management strategies reported in the literature for this rare complication.

## Case presentation

A 64-year-old woman, married and mother of eight children, living in Oujda, Morocco, was admitted for the management of rectal adenocarcinoma. Her medical history was notable for arterial hypertension treated with amlodipine 10 mg daily, and hypothyroidism managed with levothyroxine 100 µg/day for the past 10 years. She had no history of diabetes mellitus, renal disease, or cardiac disease. There was no prior surgical history and no family history of colorectal cancer or similar conditions.

The patient reported a three-month history of moderately abundant rectal bleeding associated with rectal syndrome and alternating diarrhea and constipation. Progressive worsening of symptoms prompted consultation with a gastroenterologist. Rectosigmoidoscopy revealed a circumferential ulcerative and exophytic lesion of the lower rectum. Histopathological examination of biopsy samples confirmed a moderately differentiated adenocarcinoma of the lower rectum. The patient was subsequently referred to the Regional Oncology Center of Oujda for further management. Her general condition remained preserved throughout this period.

On admission, the patient was conscious, hemodynamically and respiratory stable, with normally colored conjunctivae. Her performance status was WHO grade 1. Abdominal examination showed a soft, non-distended abdomen with no palpable masses or hernias, and no peripheral lymphadenopathy. Digital rectal examination revealed a circumferential ulcerative and budding tumor located approximately 2 cm from the anal verge, with blood noted on glove withdrawal.

Cardiovascular examination revealed normal heart sounds with no murmurs, regular rhythm, and symmetrical peripheral pulses. Pulmonary examination was unremarkable, with normal breath sounds and no added sounds.

**Figure 1:**
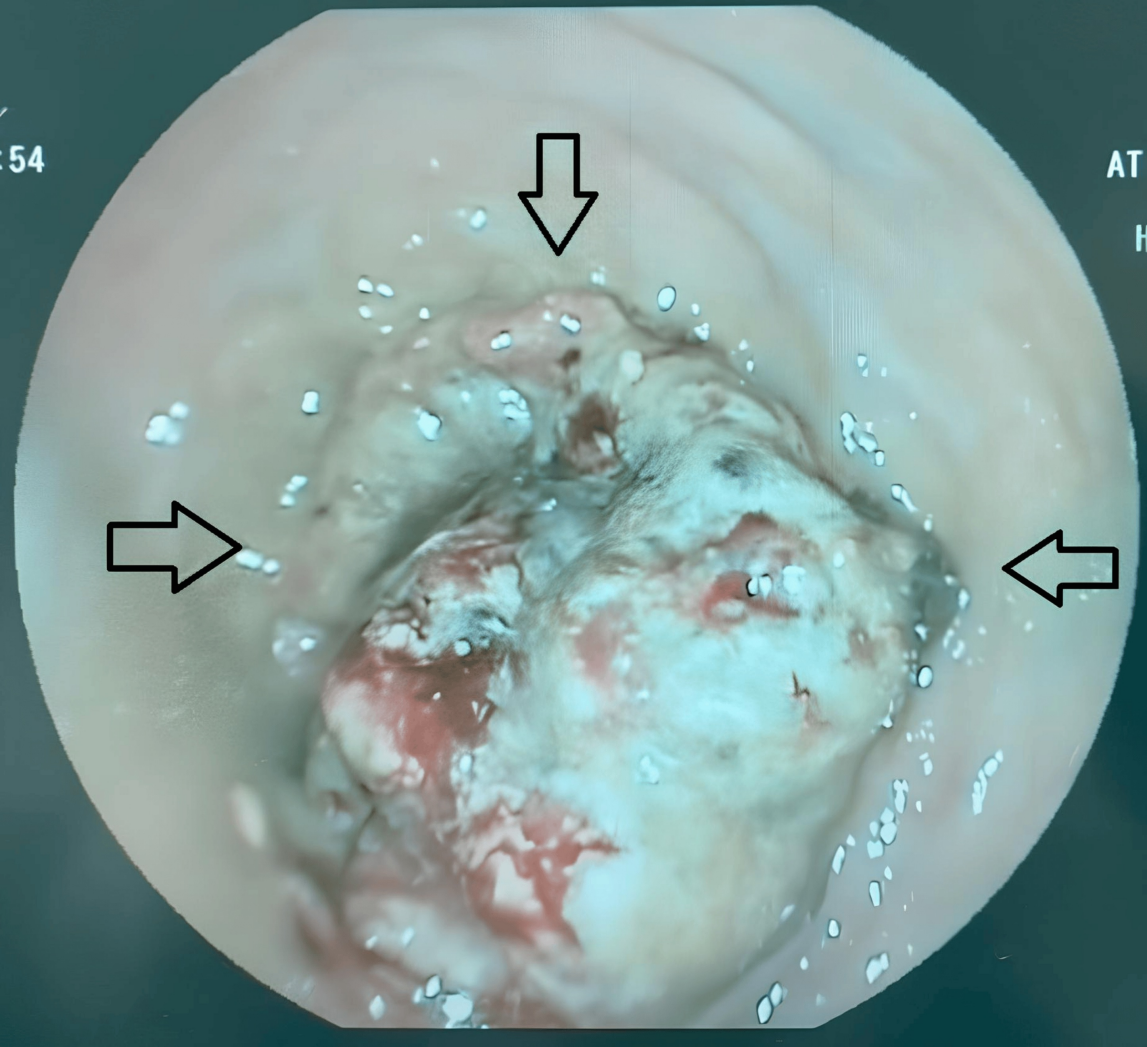
Rectosigmoidoscopy showing an ulcerofungating tumor of the lower rectum.

Rectosigmoidoscopy confirmed a circumferential ulcerative lesion of the lower rectum (Figure 1).

**Figure 2:**
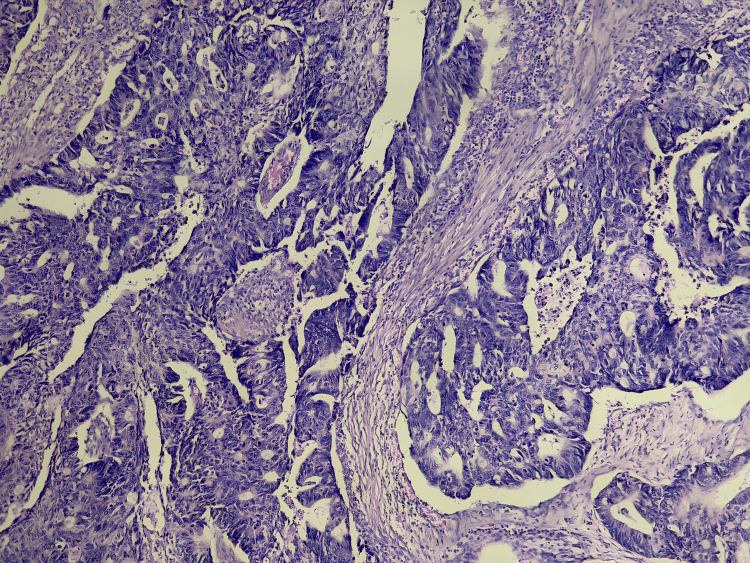
Histopathological image demonstrating a moderately differentiated adenocarcinoma of the distal rectum.

Histological analysis demonstrated a moderately differentiated infiltrating rectal adenocarcinoma (Figure 2).

**Figure 3:**
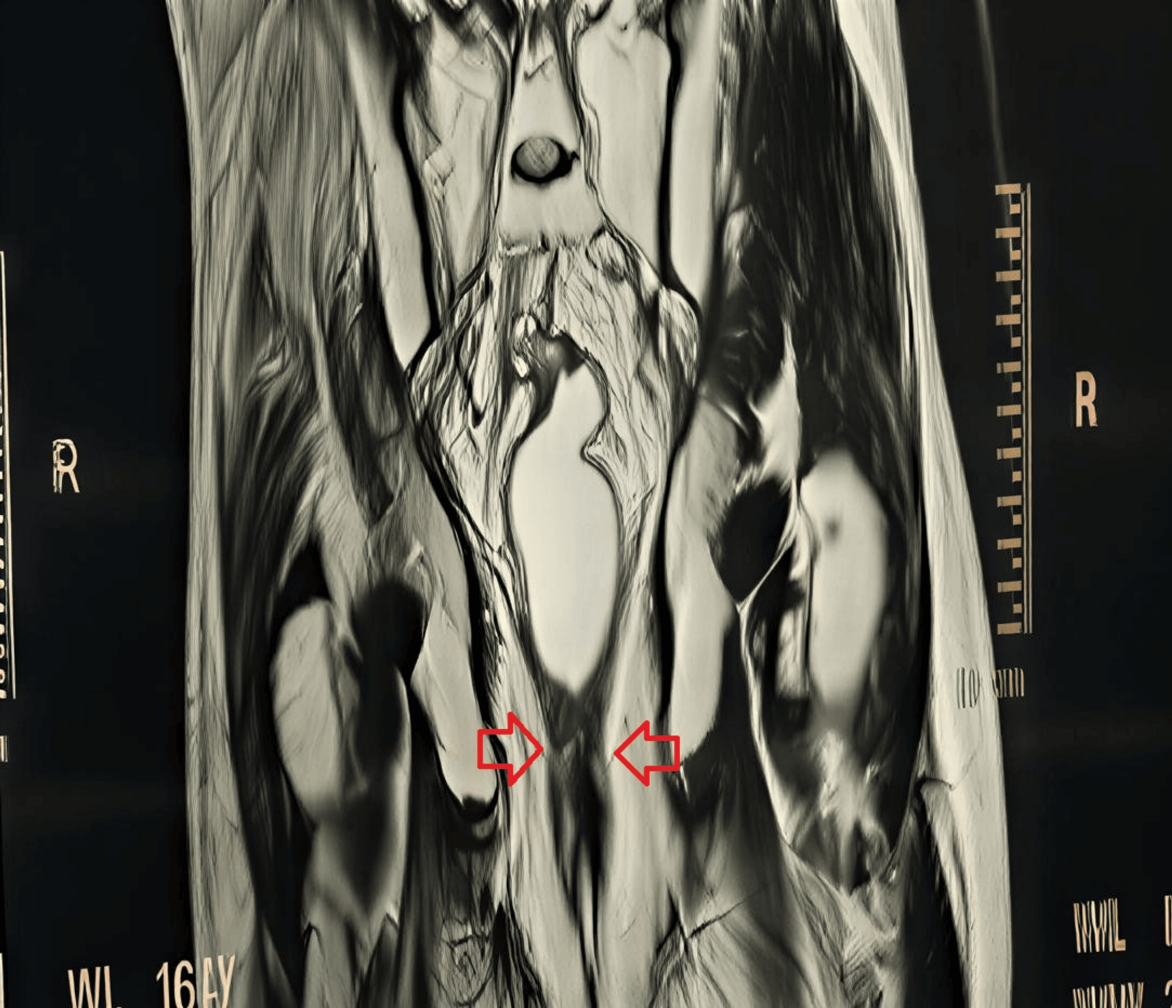
Sagittal section of pelvic magnetic resonance imaging (MRI) demonstrating a tumor of the distal rectum.

Contrast-enhanced thoraco-abdomino-pelvic computed tomography showed a lower rectal mass located 1.8 cm from the anal verge, extending over 7 cm, with mesorectal infiltration and mesorectal lymphadenopathy, without evidence of distant metastases. Pelvic magnetic resonance imaging revealed a lesion situated 1.5 cm from the anal margin, with a craniocaudal extension of 6 cm, invading the mesorectum, internal and external anal sphincters, and the levator ani muscle (Figure 3).

**Figure 4:**
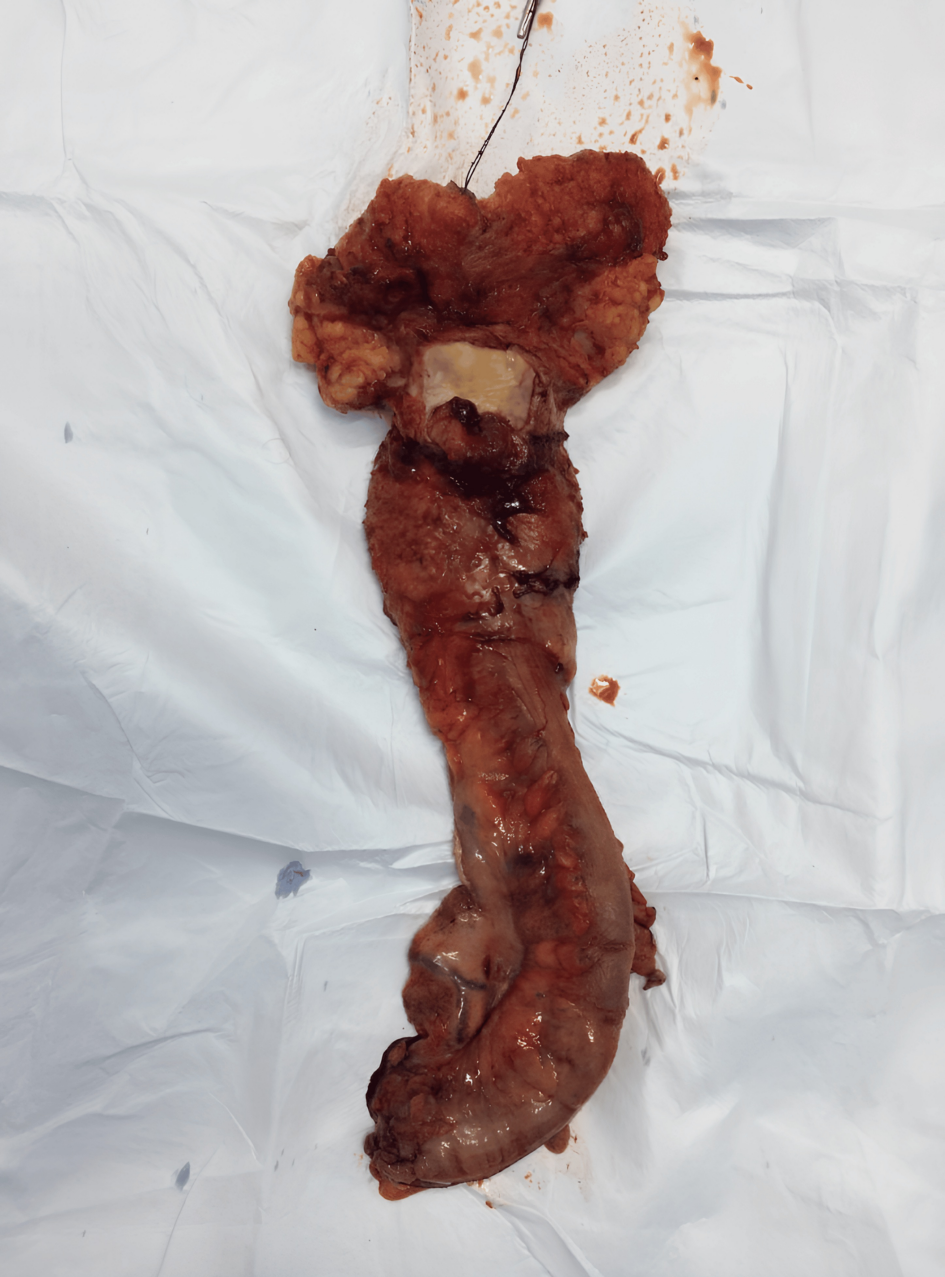
Macroscopic view of the abdominoperineal resection specimen.

The case was discussed in a multidisciplinary tumor board meeting, and the decision was made to initiate total neoadjuvant therapy (TNT) eight weeks after completion of TNT. The patient had a laparoscopic abdominoperineal resection accompanied by a terminal left iliac colostomy, in addition to the insertion of a Jackson-Pratt drain. Within our facility, we routinely apply a pelvic drain in every colorectal surgery, even in cases lacking anastomosis, like abdominoperineal amputation, to aid in the removal of any possible postoperative pelvic collections (Figure 4).

Histopathological examination of the surgical specimen showed residual moderately differentiated adenocarcinoma (Figure 2). The tumor was staged as ypT2N0Mx.

Postoperative Days 1 and 2 (POD1-POD2)

The patient remained clinically stable, with normal vital signs, no abdominal distension, and minimal serosanguinous drain output.

Postoperative Day 3 (POD3)

The patient presented with tachycardia at 120 beats/min, unexplained abdominal pain, cessation of bowel movements and gas, abdominal distension, vomiting, and marked asthenia. On clinical examination, the abdomen was distended and tender, without guarding or rigidity. Laboratory parameters showed a significant inflammatory syndrome: white blood cell count of 16,000/mm³, CRP of 180 mg/L, lactate of 1.8 mmol/L, normal electrolytes, and no fever.

**Figure 5:**
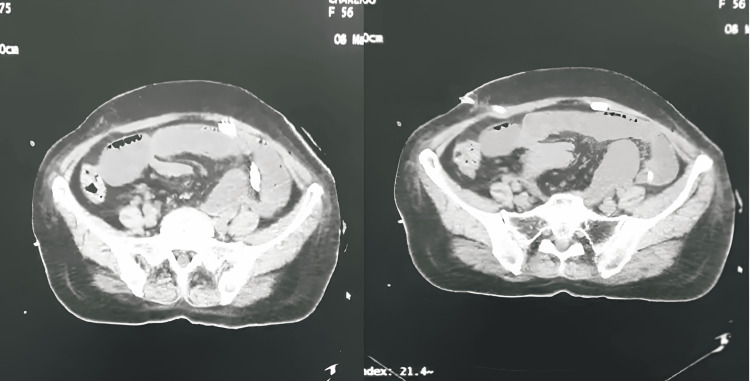
Axial slice of an abdominopelvic CT scan with intravenous contrast at the abdominal level, showing a mechanical small bowel obstruction associated with an abdominal drain.

An urgent abdominopelvic computed tomography scan demonstrated acute intestinal obstruction caused by the surgical drain (Figure 5). Removal of the drain led to resolution of the obstruction, with marked clinical amelioration.

## Discussion

Intestinal obstruction secondary to a surgical drain is a rare but potentially serious postoperative complication. While surgical site infections related to drains are frequently reported, other uncommon complications have also been described, including small bowel evisceration, bronchoperitoneal fistula, and pressure-induced intestinal perforation. Early postoperative intestinal obstruction is an uncommon issue, with a reported occurrence rate of about 0.69% in the literature. Although it is infrequent, it carries a notable mortality risk of as much as 17.8%. The risk of developing this complication largely depends on the site of the surgical procedure. Operations performed below the transverse mesocolon are associated with a significantly increased risk of intestinal obstruction, while operations confined to the upper abdomen are nearly devoid of this risk [4,5]. In our center, among 55 patients operated on for rectal adenocarcinoma in 2023, two cases of intestinal obstruction caused by an abdominal Jackson-Pratt drain were identified.

The mean age reported in the literature is 61.6 years, with extremes ranging from 30 to 82 years [4]. In our series, patients were aged 65 to 72 years. Most published studies report a clear male predominance [6,7]; however, our series showed an equal sex ratio.

Diagnosis relies on a combination of clinical and radiological findings. Clinically, intestinal obstruction classically presents with abdominal pain, cessation of stool and flatus, nausea or vomiting, and abdominal distension [2,3]. Digital rectal examination remains essential to exclude simple distal causes such as fecal impaction or to support the diagnosis when the rectal ampulla is empty [2]. The severity of obstruction depends both on the patient’s general condition and on associated radiological signs [3]. The clinical presentation of drain-related obstruction is similar to that of other postoperative intestinal obstructions, which explains the diagnostic difficulty. Several studies emphasize the need for repeated clinical examinations over a period ranging from six to 14 days to detect this complication [8,9]. In our series, patients presented with marked abdominal distension, vomiting, and complete cessation of bowel movements.

Contrast-enhanced abdominopelvic computed tomography (CT) is the reference imaging modality. It allows confirmation of intestinal obstruction, identification of the transition zone, and assessment of severity. Radiological criteria include small bowel dilatation greater than 25 mm and colonic dilatation greater than 60 mm [10] [11]. In drain-related obstructions, the causative mechanism may be identified by direct compression of a bowel loop, with a junctional zone between a dilated proximal loop and a collapsed distal loop [12,13]. The “feces sign” is a useful radiological feature, frequently visible along the drainage tract in such cases [14,15]. However, the responsibility of the drain is not always evident on imaging and may only be recognized retrospectively [16]. It is also essential to search for signs of severity such as absent bowel wall enhancement, pneumatosis intestinalis, mesenteric gas, portal venous gas, or pneumoperitoneum [4,17,18].

To date, fewer than 10 cases of intestinal obstruction caused by surgical drains have been reported in the literature (Table 1). Shah et al. described a case of small bowel obstruction caused by twisting of the intestine around a Jackson-Pratt drain following low anterior resection for rectal cancer, referred to as the “Coca-Cola mast” effect, which required small bowel resection due to ischemic necrosis. Similar cases have been reported by Poon et al. following laparoscopic colectomy [19] and by Rogers et al. after laparoscopic gastric bypass surgery [20]. Jackson-Pratt drains are most commonly implicated, although no study has formally established a correlation with a specific drain type [9]. The role of suction and negative pressure remains controversial, but it may contribute to bowel compression or herniation through drainage side holes.

**Table 1:** Summary of published cases of postoperative small bowel obstruction associated with abdominal drains in the literature.

Article / country	Year	Age	Sex	Procedure	Findings / results	Treatment
Nehme / USA	1973	72	M	Total cystectomy with ileal conduit	Compression of bowel loops by a rubber drain under negative suction	Removal of Hemovac drain tubing
Rogers / USA	2007	42	M	Gastric bypass	Small bowel loop wrapped around the abdominal drainage catheter	Catheter removal
Poon / China	2009	82	F	Anterior resection of the rectum	Small bowel herniation through the lateral holes of an intra-abdominal silicone drain, causing 90° rotation of the small intestine	Drain removal by laparoscopy
Shah / USA	2014	78	M	Anterior resection of the rectum with colorectal anastomosis and ileostomy	Small bowel wrapped around the drain	Bowel resection with anastomosis via laparotomy
Salati / Saudi Arabia	2015	62	M	Transurethral resection of the bladder dome	Mechanical compression of distal ileum by JP drain with seromuscular erosion	Exploratory laparotomy
Al Khaldi / Canada	2019	30	M	Right hepatectomy, cholecystectomy, bile duct resection with hepaticojejunostomy	Proximal jejunum wrapped around JP drain near hepaticojejunostomy	Drain removal via laparotomy
Koh / Australia	2021	39	F	Myomectomy by laparotomy	External compression of the small bowel wall	Drain removal
Bogiatzopoulos / Greece	2023	78	M	Abdominoperineal amputation	Small bowel wrapped around the drain	Drain removal after exploratory laparotomy
Nguyen / Vietnam	2023	72	M	Left colectomy	Dilated small bowel loop above the transition site	Drain removal
LIN / USA	2023	79	M	Cystectomy by laparotomy	Mechanical compression of the distal ileum by a drain	Drain removal via laparotomy

Initial management is primarily conservative in the absence of clinical or radiological severity, including bowel rest, intravenous fluid resuscitation, nasogastric decompression, and analgesia [21]. This approach may lead to the resolution of the obstruction and avoid unnecessary invasive procedures [22]. In drain-related intestinal obstruction, early removal or mobilization of the drain is a key therapeutic step and may be sufficient to relieve the obstruction [20,23]. Emergency surgical intervention is indicated in cases of clinical deterioration or radiological signs of bowel ischemia [6]. Diagnostic delay, often due to confusion with postoperative ileus, may increase patient morbidity and prolong hospitalization [10].

The most severe complication remains small bowel necrosis requiring surgical resection. These observations highlight the importance of a judicious use of prophylactic abdominal drainage. Each surgical case should be individually assessed by weighing the benefits against the risks [4]. When reoperation is required and expertise is available, laparoscopy should be considered the preferred surgical approach due to its minimally invasive nature and lower associated morbidity [24].

## Conclusions

Based on the experience of the case presented above and a review of the available literature, the following practical recommendations can be drawn: 1) Drains should only be used when deemed necessary and when the potential benefits outweigh the associated risks. 2) From the outset of the procedure, drains should be considered a possible cause of early postoperative bowel obstruction. To limit the risk of reoperation, early removal is advisable, especially in colorectal surgeries performed without anastomoses. 3) In the absence of indications justifying surgical reintervention, an obstruction due to compression of the intestinal loops by drains can generally be resolved simply by their removal, with rapid improvement in almost all cases.
